# A Systematic Approach to Time-series Metabolite Profiling and RNA-seq Analysis of Chinese Hamster Ovary Cell Culture

**DOI:** 10.1038/srep43518

**Published:** 2017-03-02

**Authors:** Han-Hsiu Hsu, Michihiro Araki, Masao Mochizuki, Yoshimi Hori, Masahiro Murata, Prihardi Kahar, Takanobu Yoshida, Tomohisa Hasunuma, Akihiko Kondo

**Affiliations:** 1 Graduate School of Science, Technology and Innovation, Kobe University, 1-1 Rokkodai, Nada, Kobe 657-8501, Japan; 2 Department of Chemical Science and Engineering, Graduate School of Engineering, Kobe University, 1-1 Rokkodai, Nada, Kobe 657-8501, Japan

## Abstract

Chinese hamster ovary (CHO) cells are the primary host used for biopharmaceutical protein production. The engineering of CHO cells to produce higher amounts of biopharmaceuticals has been highly dependent on empirical approaches, but recent high-throughput “omics” methods are changing the situation in a rational manner. Omics data analyses using gene expression or metabolite profiling make it possible to identify key genes and metabolites in antibody production. Systematic omics approaches using different types of time-series data are expected to further enhance understanding of cellular behaviours and molecular networks for rational design of CHO cells. This study developed a systematic method for obtaining and analysing time-dependent intracellular and extracellular metabolite profiles, RNA-seq data (enzymatic mRNA levels) and cell counts from CHO cell cultures to capture an overall view of the CHO central metabolic pathway (CMP). We then calculated correlation coefficients among all the profiles and visualised the whole CMP by heatmap analysis and metabolic pathway mapping, to classify genes and metabolites together. This approach provides an efficient platform to identify key genes and metabolites in CHO cell culture.

High demand for mammalian-derived biopharmaceuticals continues to stimulate the development of cell lines and bioprocess conditions. Efforts in bioprocess development have relied heavily on time-consuming and labour-intensive empirical optimisation^1^. Future progress will require a shift through knowledge of cell biology from empirical approaches to rational modification^2^,^3^,^4^. Recent developments in omics technologies have resulted in understanding host cell culture state and rational improvement of industrial mammalian cell lines by regulating growth, death and other cellular pathways through manipulation of media, feeding strategies, and other process parameters^2^.

Chinese hamster ovary (CHO) cells are the primary host used for biopharmaceutical protein production. Since the genome sequence of the CHO-K1 cell line was reported in 2011, several “omics” works have been performed to provide a knowledge base for rational engineering of CHO cells in accordance with the developmental requirements of high-throughput technology. For example, genome (Chinese hamster genome database^5^) and transcriptome (CGCDB^6^) databases were constructed for the CHO cell line. The databases triggered the development of useful CHO cell analysis pipelines, such as a CHO cell line transcript database^7^, RNA-seq differential gene expression analysis by graphical interface^8^, and development of a predictive model for productivity in CHO bioprocess culture based on gene expression profiles^9^.

Metabolite profiles measured by mass spectroscopy also provide much information for rational engineering of CHO cells. Diverse metabolic states triggered by different amino acids in antibody-producing CHO cell culture medium were analysed by poly-pathway modelling^10^. CHO metabolic behaviours resulting in physiological changes in growth and non-growth phases were analysed by *in silico* modelling, which identified pathways relevant to growth limitation, and explored major growth-limiting factors including oxidative stress and lipid metabolite depletion^11^. Moreover, isotopic tracers and mass spectrometry were used for integrative CHO cellular metabolic flux analysis, which enabled construction of a flux map for metabolic pathways such as glycolysis, the TCA cycle, lactate uptake, and the oxidative pentose phosphate pathway in different growth phases of CHO cell culture^12^.

The omics approaches mentioned above are highly dependent on data analysis to accurately process information from the high-throughput data acquisition. Software tools including Paintomics^13^, INMEX^14^, and MultiAlign^15^ were developed for transcriptomic, metabolomic, and liquid chromatography mass spectrometry (LC-MS) proteomic data analysis. Paintomics provides a web-based tool for joint visualisation of transcriptomic and metabolomic data^13^. INMEX is a web-based tool designed for analysis of multiple data sets from gene expression and metabolomic experiments^14^. MultiAlign is an efficient software package for similarity analyses searching across multiple LC-MS feature maps for both proteomic and metabolomic data^15^. The range of omics data, such as metabolite, gene expression, cell growth and culture medium profiles, is increasing, which leads to complicated interaction networks among the information from these profiles. Time-series data provide benefits for understanding cellular behaviour and molecular networks, to assist with the rational design of CHO cells. Without time-series data analysis, changes in different cell growth phases may be inadvertently ignored, and the timing of highest protein production may not be observed. Regrettably, time-series data analysis is absent from Paintomics and INMEX^13^,^14^, and although time-series analysis was used in MultiAlign, gene expression data were not included^15^. Thus, in addition to the integrated methods mentioned above^13^,^14^,^15^, systematic omics approaches producing time-series data are required to fill gaps in knowledge and to provide an overall view of CHO cells.

Here, we aim to develop a systematic time-series data analysis system, which may be used to integrate data from cell proliferation, medium supervision, mass spectrometry, and RNA-seq measurements, by calculation, heatmap analysis and metabolite mapping. CHO-K1 cells with or without lactate in the medium were cultured as an example to measure time-series for cell proliferation. The concentrations of extracellular and intracellular metabolites were measured by high-performance liquid chromatography (HPLC) and liquid chromatography with tandem mass spectrometry (LC-MS/MS). Next-generation sequencing of mRNA was performed to obtain time-series gene expression data during cell culture. We then focused on the CHO central metabolic pathway (CMP) and calculated correlation coefficients among all time-series profiles for each type of measurement. The results were integrated as heatmaps and metabolic pathway maps to comprehensively classify genes and metabolites. This approach is simple but powerful for systematic analysis of CHO cell culture with various genetic modifications to identify key genes and metabolites in multiple media or feed formulations.

## Results

### Time-series measurements of cell proliferation and extracellular glucose and lactate concentration

**Figure 1:**
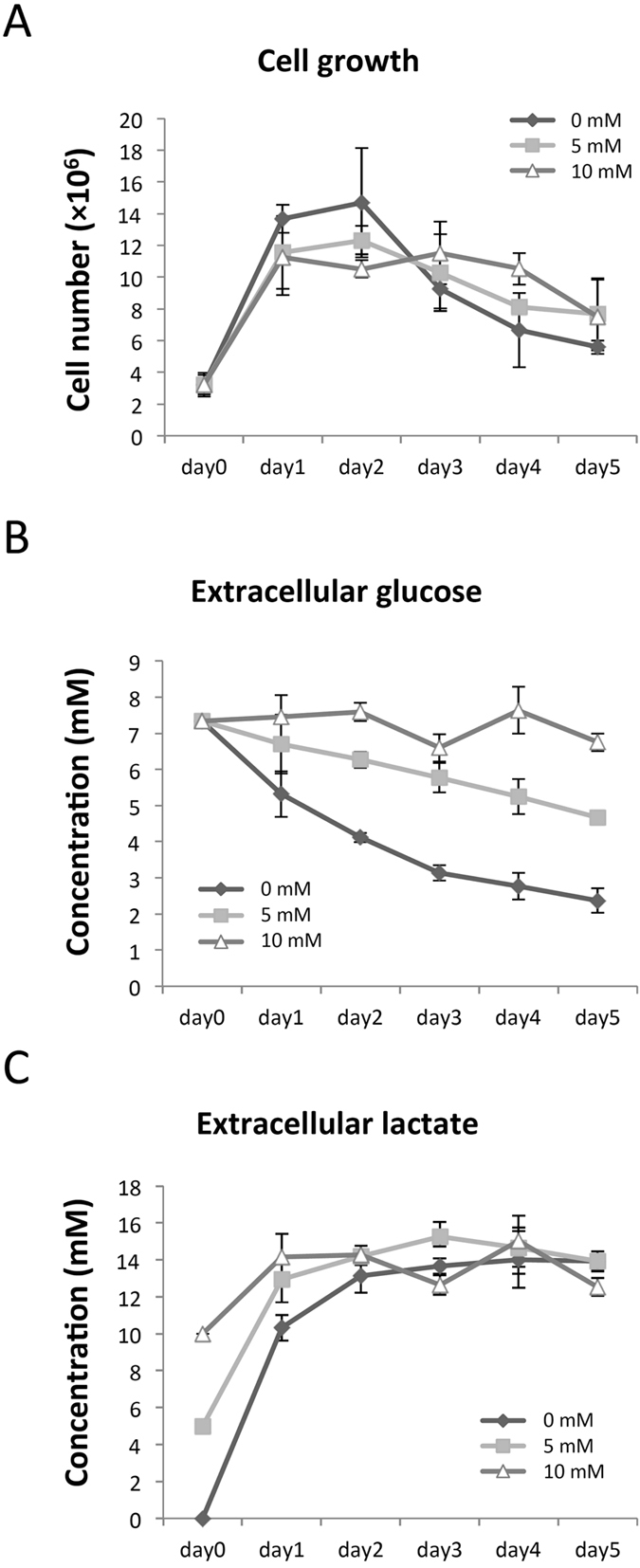
Cell number, extracellular glucose, and extracellular L-lactate measurements. CHO-K1 cell numbers were counted from day 0 to 5 (**A**) Concentrations of extracellular glucose (**B**), as well as L-lactate (**C**), were measured at the end of every 24 h from day 0 to 5 by HPLC analysis. Diamonds, squares, and triangles indicate data from the control (0 mM), 5 mM, and 10 mM lactate-containing cultures, respectively (triplicate experiments).

Time-series changes of cell number and extracellular glucose and lactate concentration were measured for cell culture in 0, 5 and 10 mM lactate-containing medium from day 0 to day 5. Cell numbers were measured by cell counting plate assay (Fig. 1A), and concentrations of extracellular glucose (Fig. 1B) and lactate (Fig. 1C) were measured by HPLC. The cell number at 0 mM lactate (control) showed faster increase from day 0 to 1 and faster decrease from day 2 to 5 compared with 5 mM and 10 mM. The maximum number of cells was observed on day 2 without lactate, and was higher than the maximum observed for 5 mM and 10 mM lactate. The differences in the maximum number of cells and the slopes of the growth curves were dependent on the amount of lactate added, so we conclude there was a definite causal relationship between cell growth and lactate addition. This tendency also held true for glucose consumption and lactate accumulation; the time-dependent changes in the concentrations of glucose and lactate were largest in the case of the control, and the shapes of the curves correlated strongly with the amount of lactate added. These results suggest that the effect of lactate addition on cell growth arose from glucose and lactate metabolism. We thus focused on the CMP, which includes glycolysis, the TCA cycle, the pentose phosphate pathway, and amino acid metabolism related to these pathways, for subsequent measurements.

### Time-series measurements of metabolites and gene expression profiles

**Figure 2:**
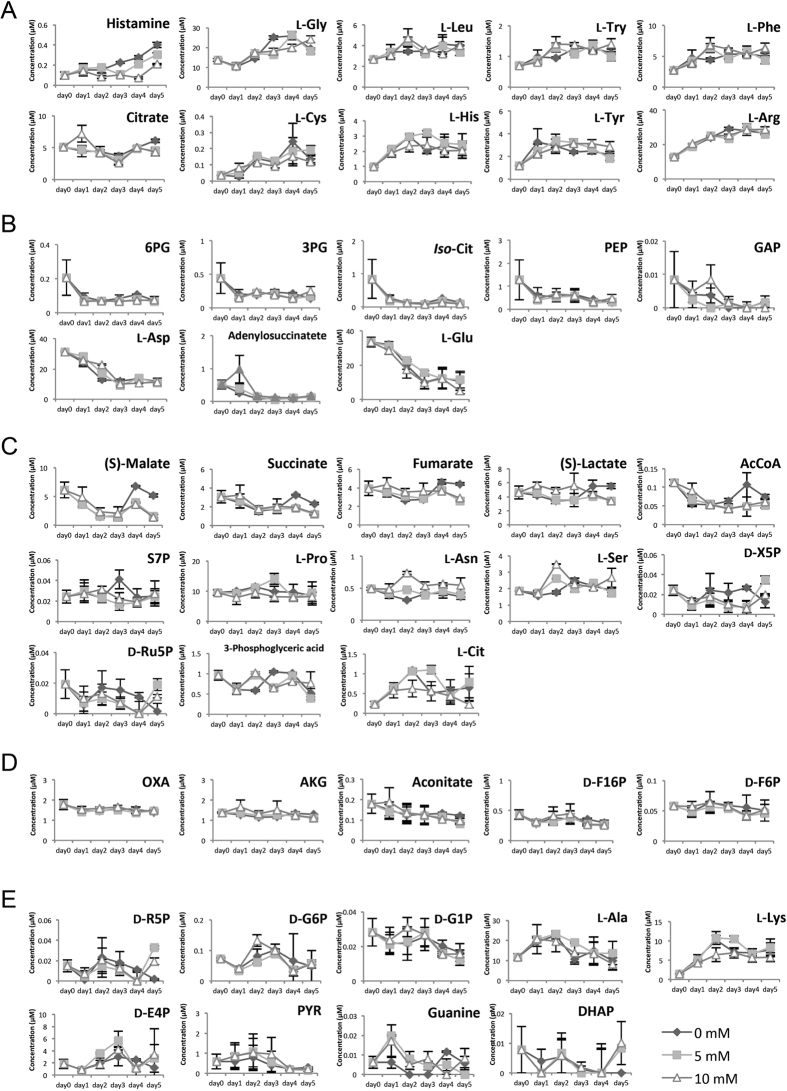
Time-series profiles of intercellular metabolites. Intracellular metabolites measured by LC-MS/MS were classified by visual inspection (**A**–**D**) Unclassified metabolites are listed in one group (**E**) Metabolite concentrations are indicated on the vertical axis (μM), and time on the horizontal axis. Diamonds, squares, and triangles indicate data from control, 5 mM, and 10 mM lactate-containing cultures, respectively (triplicate experiments). Abbreviations: 6-phospho-D-gluconate (6PG), 3-phospho-D-glycerate (3PG), *Iso*-Citrate (*Iso*-Cit), Phosphoenolpyruvate (PEP), Glyceraldehyde 3-phosphate (GAP), Acetyl-CoA (AcCoA), Sedoheptulose 7-phosphate (S7P), Oxaloacetate (OXA), 2-oxoglutarate (AKG), D-Fructose 1,6-bisphosphate (D-F16P), D-Fructose 6-phosphate (D-F6P), D-Ribose 5-phosphate (D-R5P), D-Glucose 6-phosphate (D-G6P), D-Xylulose 5-phosphate (D-X5P), D-Ribulose 5-phosphate (D-Ru5P), D-Erythrose 4-phosphate (D-E4P), pyruvate (PYR), dihydroxyacetone phosphate (DHAP), L-Serine (L-Ser), L-Asparagine (L-Asn), L-Leucine (L-Leu), L-Tryptophan (L-Try), L-Phenylalanine (L-Phe), L-Cysteine (L-Cys), D-Glucose 1-phosphate (D-G1P), L-Tyrosine (L-Tyr), L-Arginine (L-Arg), L-Aspartic acid (L-Asp), L-Glutamate (L-Glu), L-Proline (L-Pro), L-Glycine (L-Gly), L-Alanine (L-Ala), L-Citrulline (L-Cit), L-Lysine (L-Lys).

Time-series metabolite profiles (CMP and amino acid metabolism) were measured by LC-MS/MS from day 0 to 5 (Fig. 2). For quantitative measurement, we selected metabolites for which we could obtain standards and calculate the concentration of each metabolite. Metabolites involved in energy metabolism (such as ATP, ADP, AMP, NADH, NAD^+^, NADPH, and NADP^+^) were measured but not analysed in this study, because the very high amounts of these compounds (data not shown) made it difficult to compare them quantitatively with amino acids and CMP metabolites. The results showed that metabolite profiles could be classified by visual inspection into four distinct groups with respect to their temporal patterns: continuous increase (Fig. 2A), continuous decrease (Fig. 2B), variable time-series (Fig. 2C), and constant (Fig. 2D). Unclassified metabolites were listed in one group (Fig. 2E).

**Figure 3:**
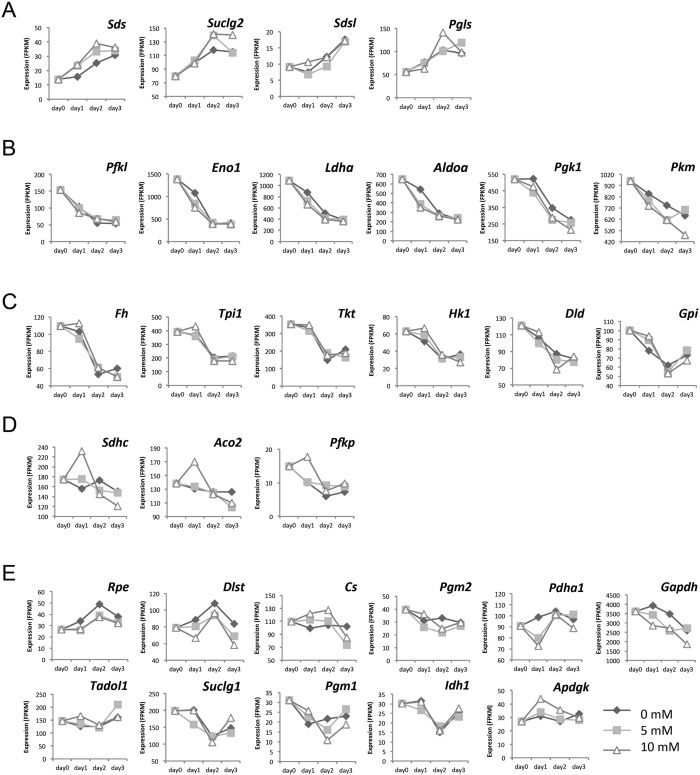
Time-series profiles of RNA-seq measurements. Time-series data from RNA-seq were classified by visual inspection (**A**–**D**) Unclassified genes are listed in one group (**E**) Gene expression levels are indicated on the vertical axis (in fragments per kilobase of transcript per million fragments, FPKM), and time on the horizontal axis. Diamonds, squares, and triangles indicate data from control, 5 mM, and 10 mM lactate-containing cultures, respectively (triplicate experiments).

Time-series gene expression profiles were measured using MiSeq from day 0 to day 3. The expression levels of genes were extracted as fragments per kilobase of exon per million mapped fragments (FPKM). Genes in CMP and amino acid metabolism were selected from the KEGG (Kyoto Encylopedia of Genes and Genomes) database by using the gene name as a keyword. In a similar manner to metabolite profiles, each gene was classified into one of four groups with respect to temporal gene expression patterns, including continuous increase (Fig. 3A), continuous decrease (Fig. 3B), fast decrease on day 2 (Fig. 3C), and high expression in 10 mM lactate on day 1 (Fig. 3D). Unclassified genes were listed in one group (Fig. 3E).

The visual classifications in Figs 2 and 3 were useful for finding metabolites and genes that may have functional links with each other. The finding that temporal patterns could be used for such classifications allowed us to move forward to overall and precise calculations in a broad, integrated way.

### Systematic correlativity analysis

**Figure 4:**
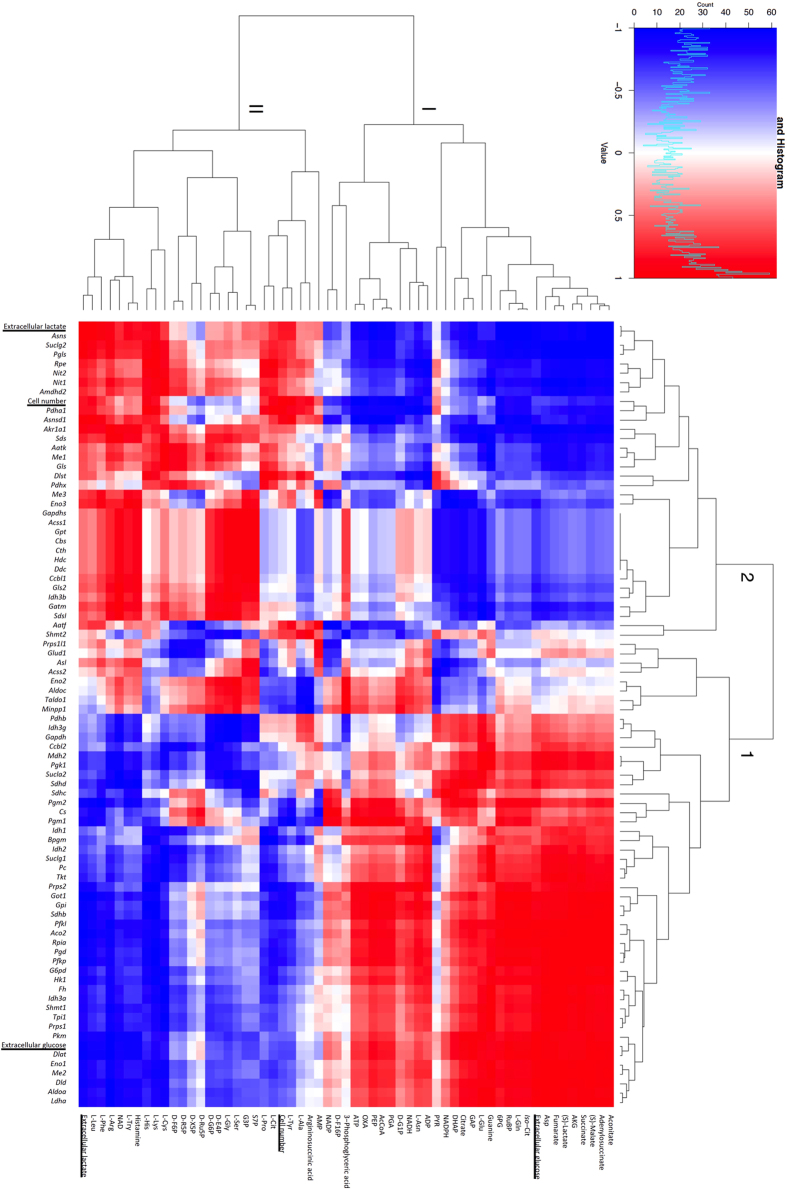
Heatmap analysis of metabolic and gene expression profiles in the control culture. Correlativity of time-series profiles of metabolites (vertical axis) and gene expression (horizontal axis) from the control culture (0 mM lactate) were calculated by Pearson’s correlation coefficient to produce cluster maps on each axis, with cell number, extracellular glucose concentration, and extracellular lactate concentration (both axes, underlined) profiles inserted. Correlativity of every metabolite and gene on the vertical axis and horizontal axis, respectively, was calculated to generate the heatmap, using programing language R. Red represents positive correlations and blue represents negative correlations. Clusters 1 and 2, and I and II, were defined for performing GO enrichment analysis (results shown in Table 1) and position analysis (results shown in Fig. 5), respectively.

Systematic analysis including metabolites and genes was expected to find causal relationships between them. To this end, we applied Pearson’s correlation coefficient to compare the time-series profiles of metabolites and gene expression. For heatmap and PathPod mapping in Figs 4 and 6, S1 and S2, time-series data from Figs 1, 2 and 3 were converted into vectors to calculate Pearson’s correlation coefficient between metabolite-metabolite, gene-gene, and metabolite-gene profiles. Metabolite profiles from the 0 mM dataset were extracted and clustered using correlativity to all other metabolite profiles. In the same way, and independently, we performed clustering using gene expression profiles. We then integrated the metabolite and gene expression profiles by calculating correlation coefficients between them. Figure 4 illustrates the integrated results in the form of a heatmap. The horizontal axis shows metabolites and the vertical axis shows genes; the distance between each metabolite or gene reflects the similarity between their profiles. We also compared time-series patterns of cell number, extracellular glucose concentration and extracellular lactate concentration to those of each metabolite and gene to identify closely related metabolites and genes (Fig. 4 underlined); red indicates positive correlation between metabolites and genes, and blue indicates negative correlation. The heatmap including metabolites, genes, cell growth, extracellular glucose, and extracellular lactate helps us to understand the overall picture of correlations among these variables. Similar heatmaps were also constructed to analyse the data for the 5 mM and 10 mM lactate-containing cultures (Figs S1 and S2).

### Metabolic pathway mapping

**Figure 5:**
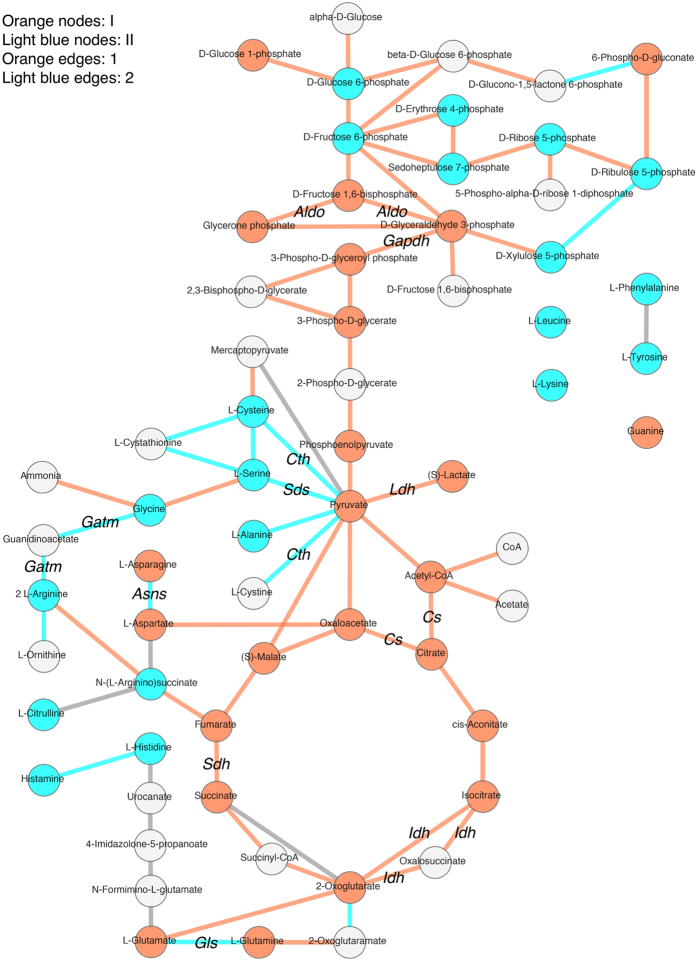
PathPod mapping for data extracted from clusters 1, 2, I, and II from data from the control culture. The positions in the metabolic pathway of each metabolite and gene in clusters 1, 2, I, and II as shown in Fig. 4 were visualised by using the PathPod mapping system. Nodes show metabolites, and edges show genes. Metabolites and genes from clusters I and 1 are tagged in orange, while those from clusters II and 2 are tagged in light blue. Metabolites and genes absent from this analysis are shown in white and grey, respectively.

Metabolites and genes in Fig. 4 were respectively placed into two large clusters (I, II, and 1, 2, respectively) on the basis of the clustering results, being the two largest clusters on each axis. To see the localisation of each metabolite and gene, we applied PathPod, a metabolic pathway mapping system developed by Araki *et al*. PathPod (http://bp.scitec.kobe-u.ac.jp/PathPod/) provides curated metabolic pathways for different organisms or cell types such as human, CHO, and yeast. Users can edit the metabolic pathway map, and plot measured data onto the edited maps using metabolite IDs and gene names. Figure 5 shows the result of mapping metabolite and gene data from the present study. Metabolite in clusters I and II are indicated by orange and light blue nodes, respectively. Genes in clusters 1 and 2 are indicated by orange and light blue edges. PathPod allows us to visualise the localisation of clusters I and II, and 1 and 2, in the form of metabolic pathways and to understand the overall picture of correlations between metabolites and genes. For example, most of the orange nodes were in glycolysis and the TCA cycle, while most of the light blue nodes were in the pentose phosphate pathway and amino acid metabolic pathways. However, most of the orange edges were in glycolysis, the pentose phosphate pathway, and the TCA cycle, while most of the light blue edges were in amino acid metabolic pathways. The results suggest that the distribution of the same colour shows functional links between metabolites and genes. In a similar way, data from the 5 mM and 10 mM lactate-containing culture analyses in Figs S1 and S2 were mapped onto PathPod, respectively (Figs S3 and S4).

### Gene Ontology enrichment analysis

From the results of PathPod mapping, the genes in clusters 1 and 2 in Fig. 4 seemed to be, respectively, functionally related. To test this idea, we analysed the genes in clusters 1 and 2 in Fig. 4 by Gene Ontology (GO) enrichment analysis (http://geneontology.org), a framework for classification of gene function and relationships. Gene IDs in each cluster were submitted for enrichment analysis for *Mus musculus* biological processes using Protein ANalysis THrough Evolutionary Relationships (PANTHER) Pathways, a classification system which curates biological databases of gene/protein families. The results are listed in Table 1. Three and six main metabolic pathways were identified for clusters 1 and 2, respectively: cluster 1 was annotated to Asn/Asp biosynthesis and the pyruvate metabolic pathways, though 20 genes were unclassified; cluster 2 was annotated to glycolysis, the TCA cycle, the pentose phosphate pathway, and pyruvate and fructose/galactose metabolic pathways, but 16 genes were unclassified (Table 1). Similar analyses were also performed using the data from Figs S3 and S4 for 5 mM and 10 mM lactate-containing cultures (Table S1). The results from GO enrichment analysis partly correspond to the PathPod mapping. GO enrichment analysis is a powerful tool to identify functions for gene clusters, but the resolution seems to be lower than that from PathPod mapping in this study, because of the absence of analysis of metabolite/gene crosstalk and metabolic network mapping.

### Comparative PathPod mapping

**Figure 6:**
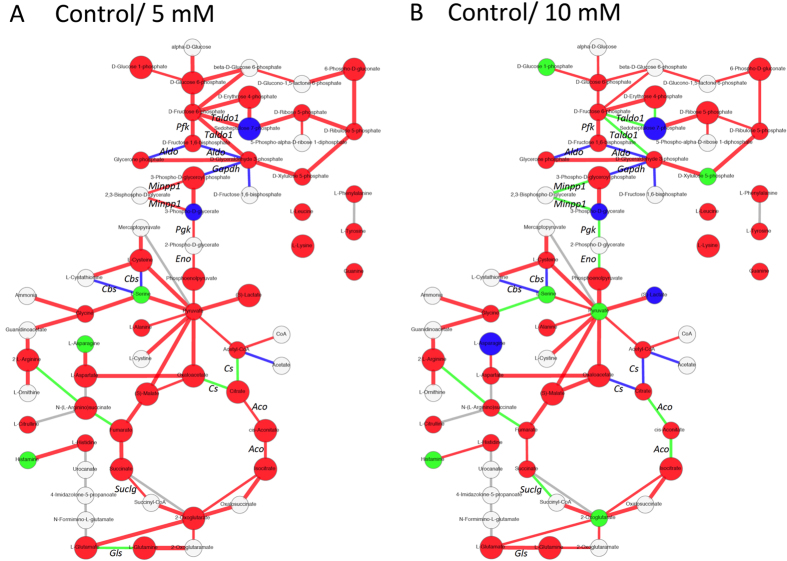
Comparative PathPod mapping system. Correlation coefficients of each metabolite and gene profile between [control (0 mM) and 5 mM lactate-containing culture] (**A**) and [0 mM and 10 mM lactate-containing culture] (**B**) were calculated and visualised using the PathPod mapping system. Each metabolite and gene is shown by a node or edge, respectively. The correlation value (R) is indicated as: bold and big in red (R > 0.9), red (0.9 > R > 0.5), green (0.5 > R > 0), blue (0 > R > −0.5), bold and big in blue (−0.5 > R). Metabolites and genes absent from this analysis are shown in white and grey, respectively. Table 2 summarises the differences between A and B according to the colour variations.

We next developed comparative PathPod mapping to test the effect of lactate addition on the metabolic pathways in CHO cells. We calculated correlation coefficients between the 0 mM and 5 mM (Fig. 6A), and between the 0 mM and 10 mM (Fig. 6B) by metabolite and gene expression profiles form LC-MS/MS and RNA-seq metrics, respectively. Each metabolite was mapped as a node in PathPod in terms of the correlation value, while each gene was mapped as an edge in PathPod in terms of the correlation value; red and blue indicate positive and negative correlations, respectively. Green indicates uncorrelated. Correlation values close to 1 and −1 are represented as big nodes for metabolites and bold edges for genes. Colours in Fig. 6A indicate the correlations between control and 5 mM lactate adding samples, and colours in Fig. 6B indicate the correlations between control and 10 mM lactate adding samples. Thus, changes in Fig. 6A and B reflect the differences between low lactate concentration and high lactate concentration, according to each of their correlations to control. In the results, the number of big red nodes and bold red edges in the control/5 mM data is greater than the number in the control/10 mM data. The observation of concentration-dependent change corresponds to the result from Fig. 1, so the differences in metabolites and genes between Fig. 6A and B may reflect the key factors affecting the CHO CMP on addition of lactate. Table 2 summarises the differences according to the colour variations. For instance, a red node in the control/5 mM data changed to a green node in control/10 mM indicates red/green in Table 2. Pyruvate is one of the red/green examples; its profile in the control was similar to that in the 5 mM growth, but was unrelated in the 10 mM growth. Similar results were observed for crucial genes that are involved in glycolysis, the TCA cycle and the pentose phosphate pathway. The comparative method can thus be a powerful tool for identifying key metabolites and genes.

## Discussion

This study developed a systematic approach for obtaining temporally resolved metabolite profiles and RNA-seq data from CHO cell culture. The approach was tested by evaluating the concentration-dependence effects of lactate on the culture process. To investigate the effect of lactate medium on cell proliferation, we focused on time-series data profiles of metabolites and genes concerned with CMP and amino acid metabolism, including glycolysis/gluconeogenesis, the TCA cycle, the pentose phosphate pathway, pyruvate metabolism, and various amino acid synthesis pathways. Time-series correlativity of the amount of each intracellular/extracellular metabolite and gene expression was calculated and illustrated with heatmaps (Figs 4, S1 and S2), and visualised by PathPod, a pathway mapping system, to identify the key factors involved in the lactate effect (Figs 5 and 6, S3 and S4).

Heatmap analysis allowed us to integrate time-series data from MS and RNA-seq together with measurements of cell number and extracellular metabolites to compare direct correlations among them. According to the calculations performed by using programming language R in Fig. 4, we demonstrated numerous of clusters on vertical and horizontal sides. Circumstances of clusters can reflect the similarities between metabolite/gene. For instance, time-series expression similarity between *Ldha* and *Aldoa* is higher than that between *Ldha* and *Cs* (Fig. 4). This could be a useful tool for comparing correlations between metabolites or genes. Moreover, to classify and localize all metabolites and genes for mapping in Fig. 5, we assigned two big clusters on each axis: clusters I and II for metabolites, and clusters 1 and 2 for genes. Although more colours could be used in Fig. 5, that is, more clusters could be assigned to each axis in Fig. 4, we suggest that using two colours is the clearest and most intuitive way for visualization in the mapping. In Fig. 4, extracellular glucose is present in cluster I, and cell number and extracellular lactate are in cluster II, while extracellular lactate and cell number are in cluster 2 and extracellular glucose is in cluster 1 (Figs 4, S1 and S2). These results indicate that time-series pattern of glucose consumption is similar in clusters I and 1, while lactate secretion and cell number variation are similar in clusters II and 2. D-Ru5P and D-X5P are in cluster II in non-lactate-containing culture (Fig. 4), and in cluster I in lactate-containing cultures (Figs S1 and S2). Pyruvate is in cluster I in non-lactate-containing culture (Fig. 4), and in cluster II in lactate-containing cultures (Figs S1 and S2). L-Asn is in cluster I in 0 mM and 5 mM lactate-containing culture (Fig. 4 and S1), and in cluster II in 10 mM lactate culture (Fig. S2). Thus, the time-series profiles of these metabolites are highly dependent on the presence of lactate, which indicated that extracellular lactate plays important roles in regulating the metabolism and amino acid flux that may relate to pharmaceutical protein production.

The distance between each metabolite or gene in the heatmap reflects the similarity in their profiles. Extracellular lactate moved closer to the cell number on the addition of lactate to the medium (Figs 4, S1 and S2), which indicates that cell proliferation is highly dependent on lactate concentration. The results corresponded with the observed lactate concentration-dependent changes in cell numbers (Fig. 1A). Furthermore, variations of the distances between extracellular glucose, extracellular lactate, and cell number and each gene and metabolite were observed in 0 mM, 5 mM, and 10 mM lactate-containing cultures. For instance, D-G1P moved closer and closer to extracellular lactate during the increase of lactate from 0 mM to 10 mM (Figs 4, S1, and S2), which indicates that intracellular D-G1P depends highly on lactate concentration in the culture medium. As lactate concentration increased, *Cs* (citrate synthase), *Gapdh* (glyceraldehyde-3-phosphate dehydrogenase) and *Sdhc* (succinate dehydrogenase) became closer to extracellular glucose, and *Sdsl* (serine dehydratase-like), *Gatm* (glycine amidinotransferase), *Idh3b* (isocitrate dehydrogenase 3 beta), and *Cth* (cystathionase) became closer to extracellular lactate and cell number (Figs 4, S1 and S2). The expression profiles of genes related to cell growth and glucose consumption were shown to be altered by changing the lactate concentration in the culture medium.

PathPod mapping was useful for comparing at a glance the extent of the effects of lactate on metabolites and genes. Metabolites in glycolysis such as D-Glucose-1-phosphate (G1P), D- fructose 1,6-bisphosphate (F16P), and 3-phosphoglyceric acid (3PG) are labelled orange (cluster I) in the control (0 mM lactate) data (Fig. 5), but light blue (cluster II) in the 10 mM lactate-containing culture (Fig. S4), which indicated that correlations between these metabolites and other metabolites were changed by the addition of 10 mM lactate to the culture medium. The results suggest that the addition of lactate led to some critical changes in CMP metabolite concentrations that affected cell proliferation. However, edge colour in PathPod mapping did not vary much among the control, 5 mM, and 10 mM lactate-containing cultures (Figs 5, S3 and S4), which suggests that the addition of lactate had less effect on gene expression in the CMP. GO enrichment analysis showed similar results: the main assigned pathways did not vary between lactate-containing cultures and the control (Table 1 and S1). From these results, it seems likely that extracellular lactate concentration directly correlated with CHO intracellular CMP metabolites, but correlated less with expression of genes in the CMP.

Comparative PathPod mapping shows that the number of big red nodes (significantly positively correlated metabolites) and bold red edges (significantly correlated genes) in the control/5 mM comparison is greater than that in the control/10 mM comparison, which suggests that the effect of lactate on metabolites and gene expression is concentration-dependent (Fig. 6). For a more detailed view, colour variations of metabolites and genes are listed in Table 2 in the form [colour in control/5 mM (Fig. 6A)]/[colour in control/10 mM (Fig. 6B)]. For example, *Gls* (encoding glutamine synthetase) showed red/green variation (changed from positively correlated to unrelated) and L-Asn showed green/blue variation (changed from unrelated to negatively correlated) in Fig. 6. The differences in colours reflect the effect of lactate. *Gls* was the only gene that changed from low correlation in control/5 mM to high correlation in control/10 mM (Fig. 6A and B). It has been reported that glutamine synthetase plays a very important role in the glutamate-glutamine cycle in the production of recombinant antibodies^16^. *Gls* knockout CHO cells are used to study improvement in CHO cell line generation efficiency^17^. Our result indicated that this gene and its protein product might be able to be regulated by the concentration of lactate feeding. We suggest this is useful information for regulating CHO cell antibody production. L-Asn was the only metabolite that changed from low correlation in 5 mM lactate to highly negative correlation in 10 mM (Fig. 6). Asparagine is reported to be the main intracellular nitrogen source related to alanine and ammonia formation, and the uptake of asparagine, together with pyruvate flux, is required to maintain TCA cycle flux for biosynthesis and energy generation^18^. Additions of glutamine and asparagine into media are reported effective on buffering pH, reducing lactate generation, maintaining cell viability, and improvement of antibody productivity by the CHO-glutamine synthetase cell line^19^. However, in our results, asparagine synthetase, *Asns*, closely tracked cell number and extracellular lactate in the heatmaps (Figs 4, S1 and S2). Thus, we suggest that *Gls* and *Asns* expression levels and the intercellular/extracellular amounts of glutamine, glutamate, and asparagine are key factors for monitoring the effect of lactate addition or production as well as in CHO cell engineering.

Other genes and metabolites listed in Table 2 may provide useful information for optimising CHO culture. For example, the product of *Gapdh*, GAPDH, is reported to functionally interact with lactate dehydrogenase, which can affect NAD^+^/NADH metabolism and glycolysis in living cells^20^. In this study, *Gapdh* was blue/blue in Fig. 6, which suggests that GAPDH is negatively sensitive to even a small amount of lactate addition. On the other hand, *Suclg* showed red/green variation, which indicated that *Suclg* genetic expression profile showed positive correlated to control in lower lactate concentration, while became unrelated to control when more lactate was added to medium. To our knowledge, this is the first study that indicated *Suclg* expression is regulated by low concentration lactate feeding. However, more experiment is needed for this issue.

Moreover, lactate and pyruvate may be fed into the medium as alternative carbon sources during cell culture in the presence of glucose^21^. In our results, pyruvate was red/green, and intracellular lactate ((S)-Lactate in Fig. 6) was red/blue (Table 2), indicating that high lactate concentration in the medium affects both of these metabolites markedly compared to low concentration. In Fig. 1, the cell number in 10 mM lactate-containing culture was higher than that in the control on day 5 (Fig. 1A), while in the latter, extracellular glucose was not really consumed (Fig. 1B) and extracellular lactate did not really accumulate (Fig. 1C). These results suggest that the presence of lactate may decrease extracellular glucose consumption, which supports the idea that lactate can be used as an alternative carbon source in late stage batch culture.

Lewis *et al*. reviewed omics approaches performed recent years and summarized their methods to define a potential framework^2^. On the other hand, Detta *et al*. reviewed bioengineering strategies of CHO cell and the impact of the knowledge gained from omics analysis^22^. According to these reviews, the purpose of most omics studies is quantifications and characterizations of various biological molecules present at a particular time and condition including genomics, transcriptomics, proteomics, and metabolomics. With omics technology, integrative analysis can be performed for identifying the metabolic and transcriptomic changes of CHO cells under different culture conditions or genomic editing, to rationally improve industrial cell line performance. However, time-series profile analysis with mapping system is rarely discussed. This study used transcriptomics and metabolomics tools described in other studies such as RNA seq, LC-MS/MS, and HPLC. What we have done more is further analysing these datasets and tried to aggregate the profiles by calculating the correlation coefficients and performing a metabolic map by using original mapping system. Moreover, we also integrated the intracellular and intercellular characteristics by using heatmap. To perform an analysis tool for time-series profiles, integrative CHO cell culture characterization by simple mapping tools is performed in this work, which is not reported in other studies.

Glycosylation plays and important role in produced biologics and biopharmaceutical industry. In this study, we focused on the effects of lactate on CMP, and the same concept of our new developed methodology here can also be used for glycosylation analysis by renewing the database and map. For this issue, analysis of metabolite profiles concern with glycosylation is also required. Database in KEGG was used in PahtPod mapping system, which provide us to introduce additional information such as metabolic and genetic profiles of glycosylation or other metabolic pathways.

The systematic analysis in this study can be further used in metabolic and transcriptomic approaches to elucidate metabolic and gene functions for bioproduction in CHO and also other cells. For example, time-series specific protein productivity profiles can be measured and inserted into heatmaps or PathPod to identify key factors. The effects of medium additives (lactate, in this study) on cell proliferation and protein production can be also analysed using the PathPod mapping system. The approach shown here thus provides powerful tools for systematic and general metabolic/transcriptomic data analysis for mammalian cell culture. Moreover, for CHO cell engineering, information such as genetic expression profiles is necessary. We suggest that analytical tools in this study may be further used for CHO cell engineering to control CMP. Analytical tools in this study could identify key factors or genes, and those information could provide hints and strategies for cell engineering tools such as gene expression, genome editing, metabolite regulations, or gene knockout. Moreover, in future studies, time-series data such as antibody titre, cell numbers, or medium ingredients can be arranged with gene expressions and metabolite profiles to perform mapping for engineered CHO cells in biopharmaceutical industrial analysis.

## Methods

### Cell culture and sample collection

The CHO-K1 (ECACC 85051005) cell line was cultured in 75T Cell Culture Flasks (BD Falcon, USA) in 37 °C CO_2_ incubator (Panasonic, Japan), in Ham’s F-12 Nutrient Mixture medium (Gibco, USA) containing 10% foetal bovine serum (Biosera, France), 100 U penicillin, and 100 μg/ml streptomycin (penicillin-streptomycin mixture, Nacalai, Japan). For lactate-containing culture, 2 × 10^6^ cells were precultured for 24 h; the end of the preculture was defined as the start of day 0. Then, the medium was changed to medium containing 0 mM (control), 5 mM, or 10 mM lactate, respectively, followed by continuous batch culture without medium change. Cell samples for metabolic and gene expression measurements were collected every 24 h from day 0 to day 5, trypsinised with 0.025% trypsin-EDTA (Gibco, USA) for 2 min at 37 °C, washed with medium once, and counted on a cell counting plate (Waken, Japan).

### Time-series extracellular measurement by HPLC

Extracellular glucose and L-lactate in culture medium were analysed by HPLC fitted with a refractive index detector (RID-10A; Shimadzu, Japan) and a photo diode array detector (SPD-M20A; Shimadzu). The ICSepICE-COREGEL-87H column was used at a flow rate of 0.6 ml/min, with the elution program consisting of an isocratic elution with 5 mM H_2_SO_4_ buffer at 80 °C for 30 min per sample. All experimental results are the averages of triplicates.

### Time-series metabolite profile measurements by LC-MS/MS

Intracellular metabolites concerned with CMP, including those from glycolysis, the TCA cycle, and the pentose phosphate pathway were analysed using a 6460 Triple Quad LC/MS (Agilent Technology, USA), and metabolites concerned with amino acid metabolism were analysed using an LCMS-8050 (Shimadzu), by reported methodology^23^ with a 150 × 2.1 mm MASTRO C18 3 μm column (Shimadzu) and a 2.1 mm I.D. × 150 mm, 3 μm Discovery HS F5-3 column (Sigma-Aldrich, USA), respectively. For cell sampling, 2 × 10^6^ CHO-K1 cells at day 0–5 were obtained and quenched with five volumes of ice-cold 150 mM NaCl (Wako, Japan), and then centrifuged at 1,300× *g* for 3 min at −5 °C. The supernatant was discarded, and the cell pellet was well suspended in 200 μl of ice-cold methanol and 100 μl of chloroform, followed by the sequential addition of 450 μl of cold methanol/3.8 mM tricine (Nacalai) (9:1) mixture and 250 μl of chloroform (Wako). Well-mixed sample solution was centrifuged at 18,000× *g* for 20 min at 0 °C, followed by transferring 300 μl of the methanol-aqueous layer into a new 1.5-ml tube. Sample extract was dried by centrifugal evaporator (EYELA, Japan) for 8 h and diluted with 100 μl of 20% methanol-water solution (v/v), followed by LC-MS/MS analysis. D-Camphorsulfonic acid (156.6 μg/l) (Nacalai) was used as an internal standard for CMP metabolite analysis, and 0.1 mM L-methionine sulfone (Sigma-Aldrich) and 10 mM 2-morpholinoethanesulfonic acid (Dojindo, Japan) were used as standards for amino acid metabolic pathway measurement. MassHunter Workstation software (Agilent) and LabSolutions (Shimadzu) were used for data analysis. Results are shown as metabolite concentration in each sample extract solution.

### cDNA library preparation and mRNA sequencing

Total RNA from cultured CHO cells was extracted by using the NucleoSpin RNA kit (Machery-Nagel, Germany), followed by quality assessment by 2100 Bioanalyzer RNA 6000 nano (Agilent) and RNA Integrity Number. Polyadenylated mRNAs were isolated with a Dynabeads mRNA DIRECT Micro Purification Kit (Thermo Fisher Scientific, USA). For each sample, a cDNA library was constructed from 25 ng of mRNA using the NEBNext Ultra Directional RNA Library Prep Kit for Illumina (New England Biolabs, USA). Distributions of the template length and adapter-dimer contamination were assessed by using a 2100 Bioanalyzer High Sensitivity DNA (Agilent). The average template length was approximately 340 bp. Contamination of adapter-dimers was ignorable. The concentration of cDNA libraries was determined using a Qubit dsDNA HS Assay Kit (Thermo Fisher), and cDNA libraries were paired-end sequenced on a MiSeq system with MiSeq Regent Kit v3 (150-cycles) (Illumina, USA).

### Sequencing data analysis

Sequencing data analysis included quality control, low quality base trimming, reference sequence mapping, and expression level calculation with CLC Genomics Workbench 8.5 (QIAGEN, Germany). Assembly and annotation of the *Cricetulus griseus* genome (GenBank assembly accession number: GCA_000419365.1) was used as a reference sequence.

### Gene Ontology enrichment analysis

Gene IDs in clusters 1 and 2 in each heatmap were submitted for GO enrichment analysis (http://geneontology.org), a framework for gene function and relationship classification by molecular function, cellular component, and biological process. Data were analysed by *M. musculus* biological process, followed by PANTHER Pathways analysis.

### Data processing and mapping

From HPLC, LC-MS/MS, and RNA-seq results, data were extracted and listed by visual inspection-based classification, according to time-series profile patterns. For heatmap and PathPod mapping, time-series data were converted into vectors to calculate Pearson’s correlation coefficient between metabolite-metabolite, gene-gene, and metabolite-gene profiles. Data were analysed and illustrated by heatmapping using the programming language R, and mapped using the PathPod mapping system (http://bp.scitec.kobe-u.ac.jp/PathPod/).

## Additional Information

**How to cite this article:** Hsu, H.-H. *et al*. A Systematic Approach to Time-series Metabolite Profiling and RNA-seq Analysis of Chinese Hamster Ovary Cell Culture. *Sci. Rep.* **7**, 43518; doi: 10.1038/srep43518 (2017).

**Publisher's note:** Springer Nature remains neutral with regard to jurisdictional claims in published maps and institutional affiliations.

## Supplementary Material

Supplementary Information

## Figures and Tables

**Table 1:** GO enrichment analysis for clusters 1 and 2 from Fig. 5.

Clusters	1		2	
Main pathway 1	Glycolysis	12	Asn/Asp biosynthesis	2
Main pathway 2	TCA cycle	6	Pyruvate metabolism	2
Main pathway 3	Pentose phosphate pathway	6	Unclassified	20
Main pathway 4	Pyruvate metabolism	4		
Main pathway 5	Fructose/Galactose metabolism	3		
Main pathway 6	Unclassified	16		

**Table 2:** Color variations in Fig. 6.

[Control/5 mM]/[Control/10 mM]	Red/Green		Green/Blue		Blue/Blue		Green/Red		Red/Blue	
Metabolite (M)/Gene (G)	M	G	M	G	M	G	M	G	M	G
	D-G1P	*Taldo1*	L-Asn	*Cs*	3PG	*Aldo*		*Gls*	(S)Lactate	
	D-X5P	*Minpp1*			S7P	*Gapdh*				
	PYR	*Eno*				*Cbs*				
	AKG	*Pgk*								
		*Aco*								
		*Suclg*								
		*Agxt*								
