# Supplementary material for: A Systematic Approach to Time-series Metabolite Profiling and RNA-seq Analysis of Chinese Hamster Ovary Cell Culture
Source: Sci Rep. 2017 Mar 2;7:43518. doi: 10.1038/srep43518 (PMC5333161; doi:10.1038/srep43518)
Supplement: Supplementary Information [file srep43518-s1.pdf]

Supplementary Info File

A Systematic Approach to Time-series Metabolite Profiling and RNA-seq Analysis of Chinese Hamster Ovary Cell Culture

Han-Hsiu Hsu[1], Michihiro Araki[1], Masao Mochizuki[1], Yoshimi Hori[1], Masahiro Murata[1], Prihardi Kahar[2], Takanobu Yoshida[1], Tomohisa Hasunuma[1], Akihiko Kondo[1,2*]

[1]Graduate School of Science, Technology and Innovation, Kobe University, 1-1 Rokkodai, Nada, Kobe 657-8501, Japan

[2]Department of Chemical Science and Engineering, Graduate School of Engineering, Kobe University, 1-1 Rokkodai, Nada, Kobe 657-8501, Japan

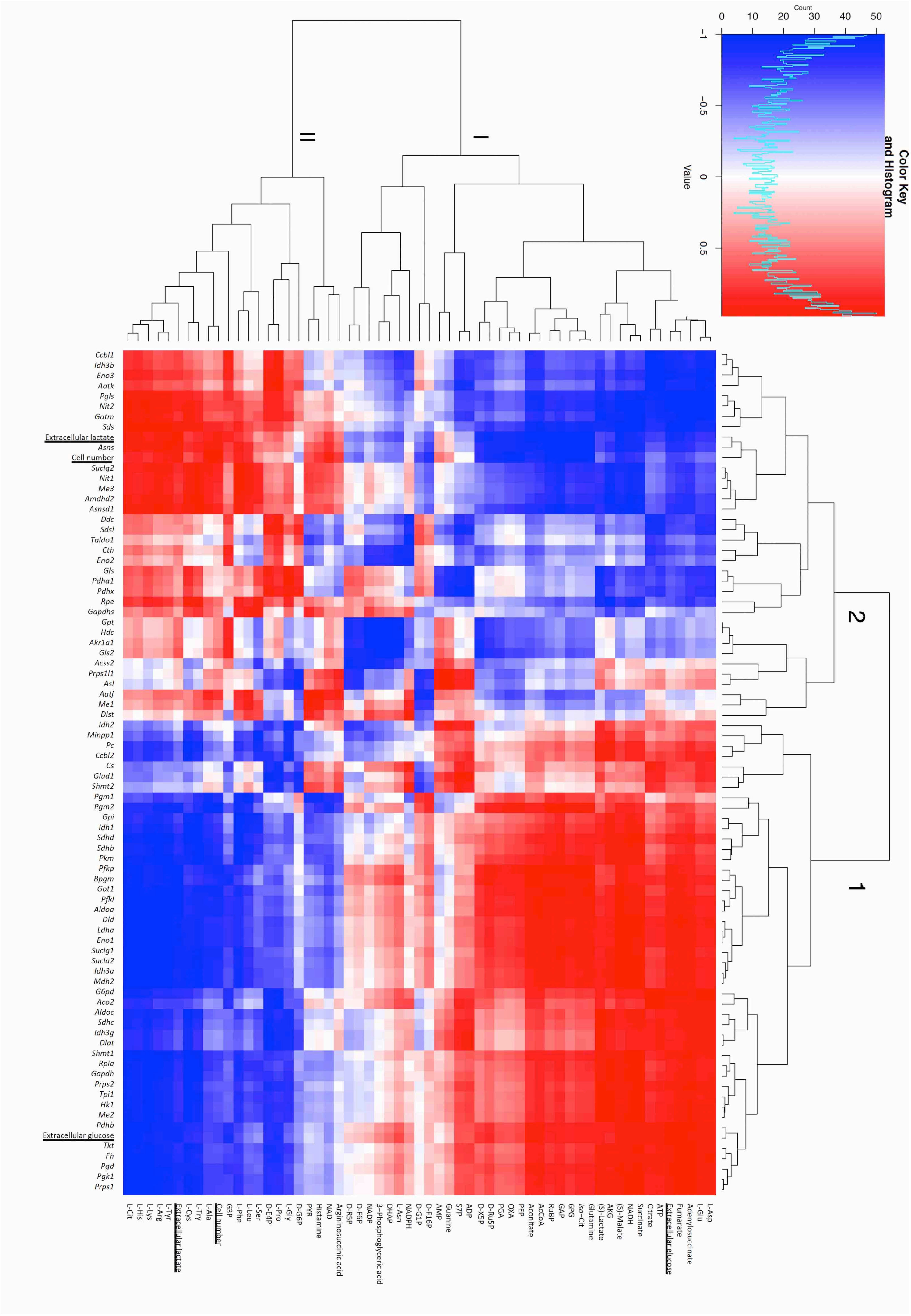

Color Key and Histogram
Count
0
10
20
30
40
50
Value
−1
−0.5
0
0.5
II
I
2
1
Ccbl1
Idh3b
Eno3
Aatk
Pgls
Nit2
Gatm
Sds
Extracellular lactate
Asns
Cell number
Suclg2
Nit1
Me3
Amdhd2
Asnsd1
Ddc
Sdsl
Taldo1
Cth
Eno2
Gls
Pdha1
Pdhx
Rpe
Gapdhs
Gpt
Hdc
Akr1a1
Gls2
Acss2
Prps1l1
Asl
Aatf
Me1
Dlst
Idh2
Minpp1
Pc
Ccbl2
Cs
Glud1
Shmt2
Pgm1
Pgm2
Gpi
Idh1
Sdhd
Sdhb
Pkm
Pfkp
Bpgm
Got1
Pfkl
Aldoa
Dld
Ldha
Eno1
Suclg1
Sucla2
Idh3a
Mdh2
G6pd
Aco2
Aldoc
Sdhc
Idh3g
Dlat
Shmt1
Rpia
Gapdh
Prps2
Tpi1
Hk1
Me2
Pdhb
Extracellular glucose
Tkt
Fh
Pgd
Pgk1
Prps1
L-Cit
L-His
L-Lys
L-Arg
L-Tyr
Extracellular lactate
L-Cys
L-Try
L-Ala
Cell number
G3P
L-Phe
L-Leu
L-Ser
D-E4P
L-Pro
L-Gly
D-G6P
PYR
Histamine
NAD
Argininosuccinic acid
D-R5P
D-F6P
NADP
3-Phosphoglyceric acid
DHAP
L-Asn
NADPH
D-G1P
D-F16P
AMP
Guanine
S7P
ADP
D-X5P
D-Ru5P
PGA
OXA
PEP
Aconitate
AcCoA
RuBP
GAP
6PG
Iso-Cit
Glutamine
(S)-Lactate
AKG
(S)-Malate
NADH
Succinate
Citrate
ATP
Extracellular glucose
Fumarate
Adenylosuccinate
L-Glu
L-Asp

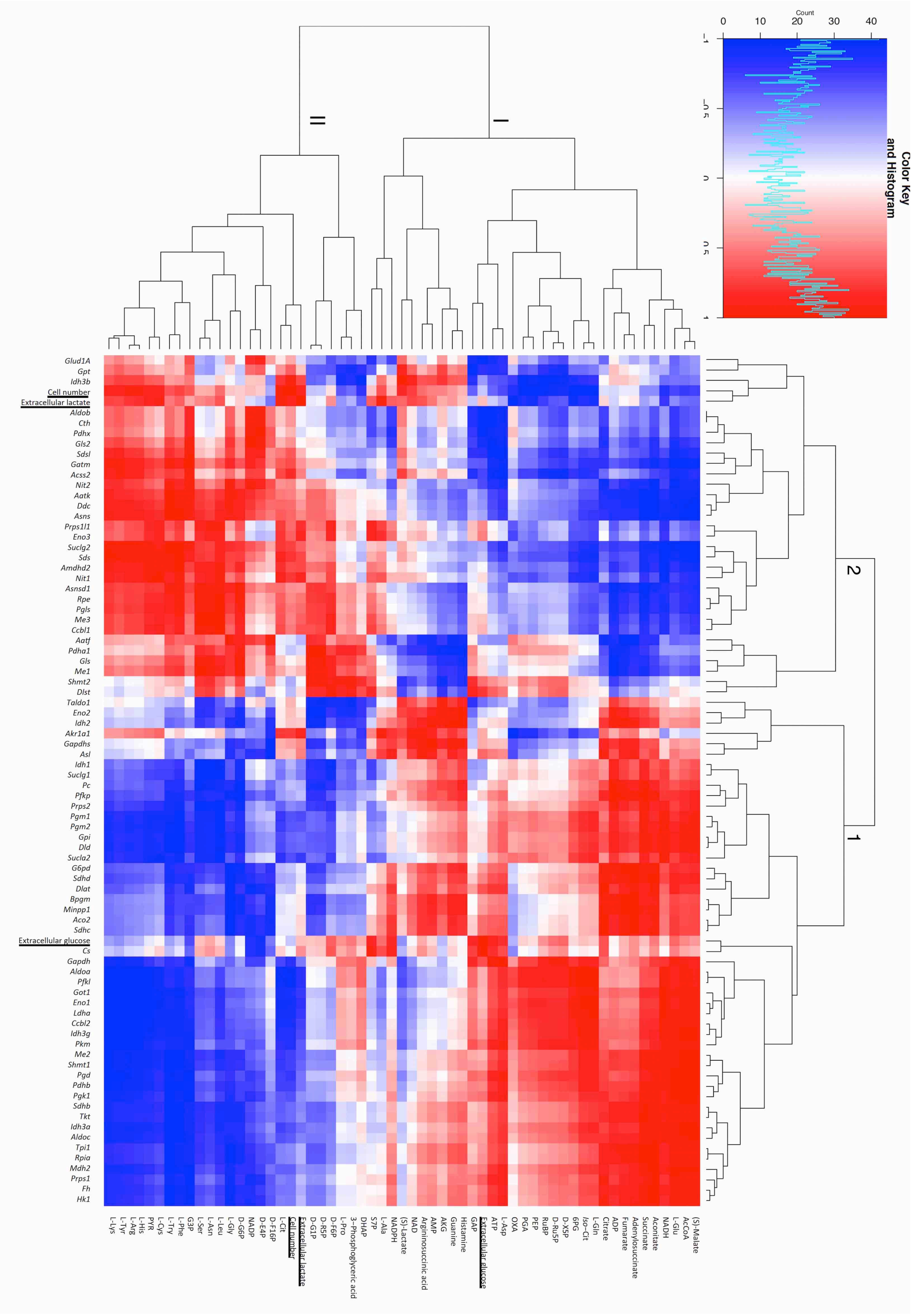

Color Key and Histogram
Count
0
10
20
30
40
II
I
2
1
Glud1A
Gpt
Idh3b
Cell number
Extracellular lactate
Aldob
Cth
Pdhx
Gls2
Sdsl
Gatm
Acss2
Nit2
Aatk
Ddc
Asns
Prps1l1
Eno3
Suclg2
Sds
Amdhd2
Nit1
Asnsd1
Rpe
Pgls
Me3
Ccbl1
Aatf
Pdha1
Gls
Me1
Shmt2
Dlst
Taldo1
Eno2
Idh2
Akr1a1
Gapdhs
Asl
Idh1
Suclg1
Pc
Pfkp
Prps2
Pgm1
Pgm2
Gpi
Dld
Sucla2
G6pd
Sdhd
Dlat
Bpgm
Minpp1
Aco2
Sdhc
Extracellular glucose
Cs
Gapdh
Aldoa
Pfkl
Got1
Eno1
Ldha
Ccbl2
Idh3g
Pkm
Me2
Shmt1
Pgd
Pdhb
Pgk1
Sdhb
Tkt
Idh3a
Aldoc
Tpi1
Rpia
Mdh2
Prps1
Fh
Hk1
L-Lys
L-Tyr
L-Arg
L-His
PYR
L-Cys
L-Try
L-Phe
G3P
L-Ser
L-Asn
L-Leu
L-Gly
D-G6P
NADP
D-E4P
D-F16P
L-Cit
Cell number
Extracellular lactate
D-G1P
D-R5P
D-F6P
L-Pro
3-Phosphoglyceric acid
DHAP
S7P
L-Ala
NADPH
(S)-Lactate
NAD
Argininosuccinic acid
AMP
AKG
Guanine
Histamine
GAP
Extracellular glucose
ATP
L-Asp
OXA
PGA
PEP
RuBP
D-Ru5P
D-X5P
6PG
Iso-Cit
L-Gln
Citrate
ADP
Fumarate
Adenylosuccinate
Succinate
Aconitate
NADH
L-Glu
AcCoA
(S)-Malate

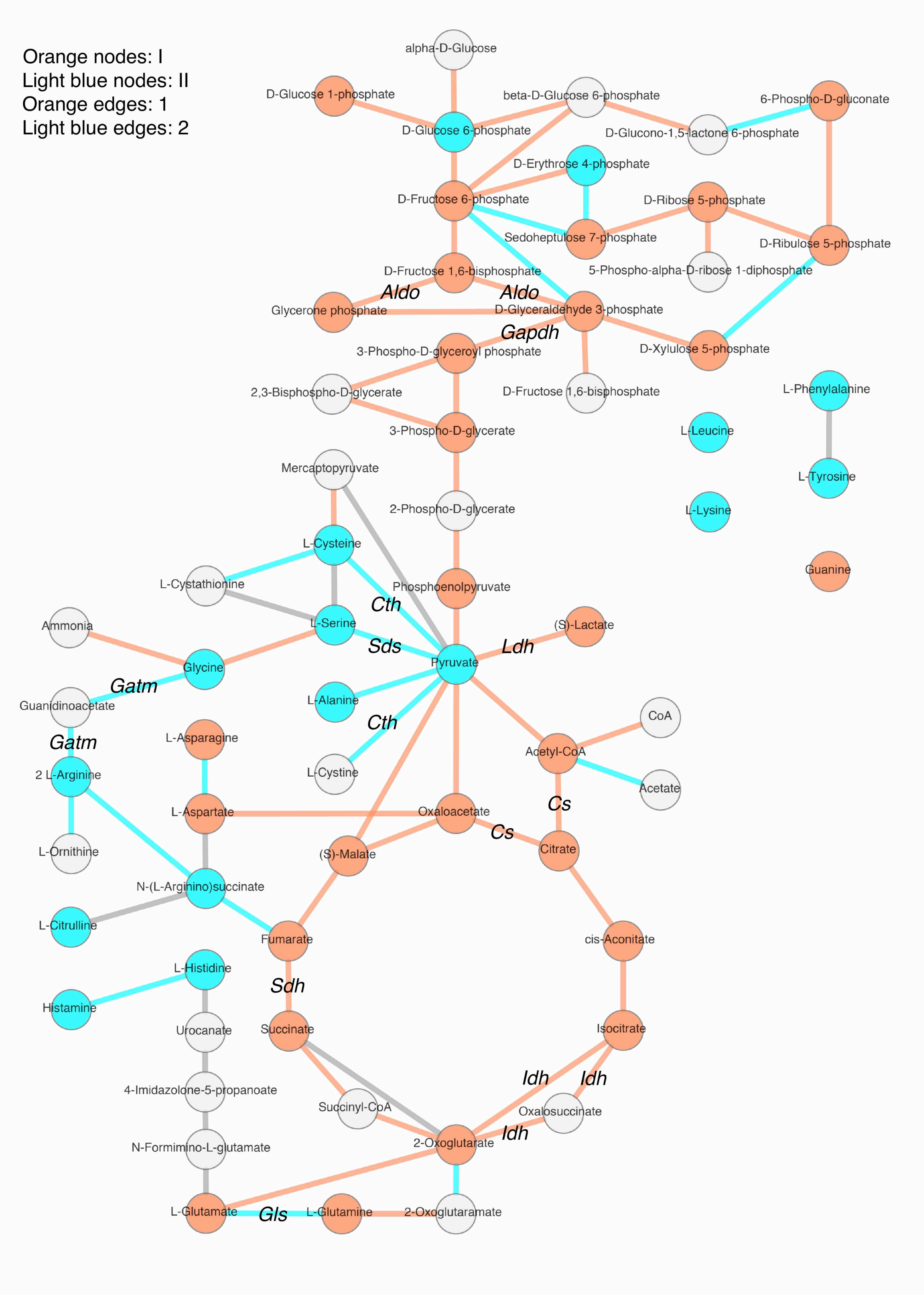
Orange nodes: I
Light blue nodes: II
Orange edges: 1
Light blue edges: 2
alpha-D-Glucose
D-Glucose 1-phosphate
beta-D-Glucose 6-phosphate
6-Phospho-D-gluconate
D-Glucose 6-phosphate
D-Glucono-1,5-lactone 6-phosphate
D-Erythrose 4-phosphate
D-Fructose 6-phosphate
D-Ribose 5-phosphate
Sedoheptulose 7-phosphate
D-Ribulose 5-phosphate
D-Fructose 1,6-bisphosphate
5-Phospho-alpha-D-ribose 1-diphosphate
Aldo
Aldo
Glycerone phosphate
D-Glyceraldehyde 3-phosphate
Gapdh
3-Phospho-D-glyceroyl phosphate
D-Xylulose 5-phosphate
2,3-Bisphospho-D-glycerate
D-Fructose 1,6-bisphosphate
L-Phenylalanine
3-Phospho-D-glycerate
L-Leucine
Mercaptopyruvate
L-Tyrosine
2-Phospho-D-glycerate
L-Lysine
L-Cysteine
Guanine
L-Cystathionine
Phosphoenolpyruvate
Cth
Ammonia
L-Serine
(S)-Lactate
Sds
Ldh
Glycine
Pyruvate
Gatm
Guanidinoacetate
L-Alanine
Cth
CoA
Gatm
L-Asparagine
Acetyl-CoA
2 L-Arginine
L-Cystine
Acetate
Cs
L-Aspartate
Oxaloacetate
Cs
L-Ornithine
(S)-Malate
Citrate
N-(L-Arginino)succinate
L-Citrulline
Fumarate
cis-Aconitate
L-Histidine
Sdh
Histamine
Urocanate
Succinate
Isocitrate
4-Imidazolone-5-propanoate
Idh
Idh
Succinyl-CoA
Oxalosuccinate
Idh
N-Formimino-L-glutamate
2-Oxoglutarate
L-Glutamate
Gls
L-Glutamine
2-Oxoglutaramate

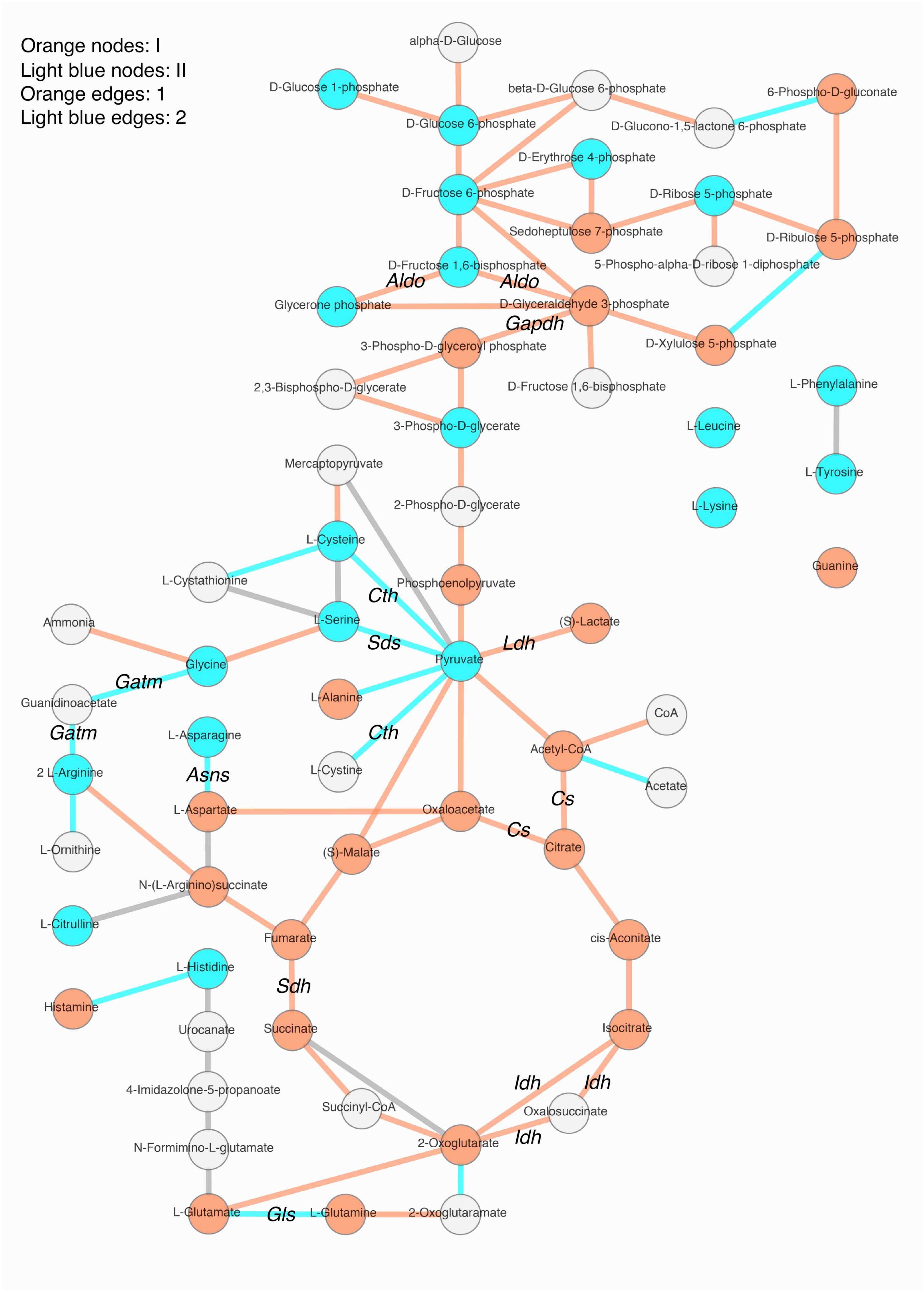
Orange nodes: I
Light blue nodes: II
Orange edges: 1
Light blue edges: 2
alpha-D-Glucose
D-Glucose 1-phosphate
beta-D-Glucose 6-phosphate
6-Phospho-D-gluconate
D-Glucose 6-phosphate
D-Glucono-1,5-lactone 6-phosphate
D-Erythrose 4-phosphate
D-Fructose 6-phosphate
D-Ribose 5-phosphate
Sedoheptulose 7-phosphate
D-Ribulose 5-phosphate
D-Fructose 1,6-bisphosphate
5-Phospho-alpha-D-ribose 1-diphosphate
Aldo
Aldo
Glycerone phosphate
D-Glyceraldehyde 3-phosphate
Gapdh
3-Phospho-D-glyceroyl phosphate
D-Xylulose 5-phosphate
2,3-Bisphospho-D-glycerate
D-Fructose 1,6-bisphosphate
L-Phenylalanine
3-Phospho-D-glycerate
L-Leucine
Mercaptopyruvate
L-Tyrosine
2-Phospho-D-glycerate
L-Lysine
L-Cysteine
Guanine
L-Cystathionine
Phosphoenolpyruvate
Cth
Ammonia
L-Serine
(S)-Lactate
Sds
Ldh
Pyruvate
Glycine
Gatm
Guanidinoacetate
L-Alanine
CoA
Gatm
L-Asparagine
Cth
Acetyl-CoA
2 L-Arginine
L-Cystine
Asns
Acetate
L-Aspartate
Oxaloacetate
Cs
Cs
Citrate
L-Ornithine
(S)-Malate
N-(L-Arginino)succinate
L-Citrulline
Fumarate
cis-Aconitate
L-Histidine
Sdh
Histamine
Urocanate
Succinate
Isocitrate
4-Imidazolone-5-propanoate
Idh
Idh
Succinyl-CoA
Oxalosuccinate
N-Formimino-L-glutamate
2-Oxoglutarate
Idh
L-Glutamate
Gls
L-Glutamine
2-Oxoglutaramate

Table S1 GO analysis results of Figs. S1 and S2

5 mM

Clusters	1		2	
**Main pathway 1**	Glycolysis	11	Asn/Asp biosynthesis	2
**Main pathway 2**	TCA cycle	6	Pyruvate metabolism	2
**Main pathway 3**	Pyruvate metabolism	4	Unclassified	20
**Main pathway 4**	Fructose/Galactose metabolism	3		
**Main pathway 5**	Pentose phosphate pathway	2		
**Main pathway 6**	Unclassified	15		

10 mM

Clusters	1		2	
**Main pathway 1**	Glycolysis	13	Asn/Asp biosynthesis	2
**Main pathway 2**	TCA cycle	6	Pyruvate metabolism	2
**Main pathway 3**	Pyruvate metabolism	4	Unclassified	20
**Main pathway 4**	Pentose phosphate pathway	6		
**Main pathway 5**	Fructose/Galactose metabolism	3		
**Main pathway 6**	Unclassified	16		

Figure Legends

Figure S1. Heatmap analysis of metabolic and gene expression profiles in the 5 mM lactate-containing culture. Correlativity of time-series profiles of metabolites (vertical axis) and gene expression (horizontal axis) from the 5 mM lactate-containing culture were calculated by Pearson's correlation coefficient to produce cluster maps on each axis, with cell number, extracellular glucose concentration, and extracellular lactate concentration (both axes, underlined) profiles inserted. Correlativity of every metabolite and gene on the vertical axis and horizontal axis, respectively, was calculated to generate the heatmap, using programing language R. Red represents positive correlations and blue represents negative correlations. Clusters 1 and 2, and I and II, were defined by GO enrichment analysis (results shown in Table S1), and position analysis (results shown in Fig. S3), respectively.

Figure S2. Heatmap analysis of metabolic and gene expression profiles in the 10 mM lactate-containing culture. Correlativity of time-series profiles of metabolites (vertical axis) and gene expression (horizontal axis) from the 10 mM lactate-containing culture were calculated by Pearson's correlation coefficient to produce cluster maps on each axis, with cell number, extracellular glucose concentration, and extracellular lactate concentration (both axes, underlined) profiles inserted. Correlativity of every metabolite and gene on the vertical axis and horizontal axis, respectively, was calculated to generate the heatmap, using programing language R. Red represents positive correlations and blue represents negative correlations. Clusters 1 and 2, and I and II, were defined by GO enrichment analysis (results shown in Table S1), and position analysis (results shown in Fig. S4), respectively.

Figure S3. PathPod mapping for data extracted from clusters 1, 2, I, and II from data from the 5 mM lactate-containing culture. The positions in the metabolic pathway of each

metabolite and gene in clusters 1, 2, I, and II as shown in Fig. S1 were visualised using the PathPod mapping system. Nodes indicate metabolites, and edges indicate genes. Metabolites and genes from clusters I and 1 are indicated in orange, and those from clusters II and 2 are indicated in light blue. Metabolites and genes absent from this analysis are indicated in white and grey, respectively.

Figure S4. PathPod mapping for data extracted from clusters 1, 2, I, and II from data from the 10 mM lactate-containing culture. The positions in the metabolic pathway of each metabolite and gene in clusters 1, 2, I, and II as shown in Fig. S2 were visualised using the PathPod mapping system. Nodes indicate metabolites, and edges indicate genes. Metabolites and genes from clusters I and 1 are indicated in orange, and those from clusters II and 2 are indicated in light blue. Metabolites and genes absent from this analysis are indicated in white and grey, respectively.

Table S1. GO enrichment analysis for clusters 1 and 2 in Figs. S3 and S4.
